# Adherence to rehabilitation and home exercise after myocardial infarction: a qualitative study of expectations, barriers and drivers

**DOI:** 10.1186/s13102-023-00714-3

**Published:** 2023-08-09

**Authors:** Nina Serves, Lionel Pazart, Damien Gabriel, Laurent Mourot, Fiona Ecarnot

**Affiliations:** 1 Inserm CIC1431, Centre Hospitalier Universitaire de Besançon, Besançon, 25000 France; 2 UR LINC, Université de Franche-Comté, Besançon, 25000 France; 3 Exercise Performance Health Innovation (EPHI), Université de Franche-Comté, Besançon, 25000 France; 4 EA3920, Université de Franche-Comté, Besançon, 25000 France; 5 Department of Cardiology, University Hospital Besançon, Boulevard Fleming, Besançon, 25000 France

**Keywords:** Rehabilitation, Exercise, Secondary prevention, Acute myocardial infarction

## Abstract

**Background:**

Cardiac rehabilitation is a key component of secondary prevention, but uptake is often low, and motivation to pursue exercise and lifestyle changes may be lacking in patients who have suffered from acute myocardial infarction (AMI). We explored the intentions of patients hospitalized for AMI regarding attendance at cardiac rehabilitation and the future pursuit of regular physical exercise at home.

**Methods:**

We performed a qualitative study using semi-structured interviews. Eligible patients were those hospitalized for AMI in the cardiology unit of a large university hospital in Eastern France between 10/11/2021 and 7/3/2022, and who were deemed eligible for rehabilitation by the treating physician. Patients were interviewed before discharge. Interviews were transcribed and analysed by thematic analysis. We administered the Global Physical Activity Questionnaire (GPAQ) questionnaire to all participants.

**Results:**

Of 17 eligible patients, 15 were interviewed, at which point saturation was reached. The majority were males (n = 13, 86%), median age 54 years (41–61). Three key themes emerged: Firstly, there is a mismatch between patients’ perceptions of their physical activity and actual level of activity as assessed by objective tools. Second, cardiac rehabilitation is seen as a vector for information about the return to home after AMI. Third, regarding the intention to change lifestyle, there are persisting obstacles, drivers, fears and expectations.

**Conclusion:**

Patients with AMI often overestimate how physically active they are. Even close to discharge, patients have persisting informational needs, and many see cardiac rehabilitation as a means to obtain this information, rather than as a therapeutic intervention.

## Introduction

Cardiovascular disease is the leading cause of mortality in the world, and coronary artery disease, including acute myocardial infarction, is the primary component of overall cardiovascular mortality, accounting for around 7 million deaths worldwide every year [1]. Among patients who have suffered from acute myocardial infarction (AMI), cardiac rehabilitation is recommended as a key component of secondary prevention [2]. Cardiac rehabilitation focuses in large part on physical exercise, but many patients do not exercise regularly in their daily lives. For these patients, a return to (or initiation of) regular physical exercise can imply making long-term changes to their lifestyle, in order to reap the proven benefits of exercise in terms of cardiovascular health and prevention of recurrent AMI [3].

Yet, despite its proven benefits, cardiac rehabilitation is under-utilized [4, 5]. One European study reported that less than half (45%) of patients discharged from hospital after AMI were referred for cardiac rehabilitation and only 34% actually participated [6]. Similarly, a recent study by Winnige et al. reported that only 15 to 30% of eligible patients actually attend a cardiac rehabilitation programme [3]. The main reason for non-participation is reportedly the failure to orient patients directly to rehabilitation at discharge after their AMI. Other reasons for non-referral could include patient-related characteristics, or difficulties with access to rehabilitation [7], a failure (by the patient or the physician) to perceive the benefits of rehabilitation [8] and/or of exercise in particular [9]. In addition to low referral rates, a recent review identified other barriers to participation in cardiac rehabilitation, including gender and racial/ethnic disparities, poor physical health, language barriers, the cost of rehabilitation, and long travel distance [10]. Furthermore, a significant proportion of those who are oriented to rehabilitation do not complete the full scheduled programme. Indeed, Brouwers et al. reported that around one quarter of patients who register do not successfully complete the full programme [7]. Similarly, in a meta-analysis, Turk-Adawi et al. reported that fewer than 50% of eligible patients participated in rehabilitation and drop-out rates ranged from 12 to 56% in high-income countries, while participation rates were as low as < 30% in some reports from middle- and lower-income countries, with drop-out rates as high as 82% in studies from Iran [11].

Once they return home, regardless of whether or not they attended rehabilitation, a majority of post-AMI patients do not take up (or continue) regular physical exercise, with 26% of post-MI patients remaining constantly inactive or less active than prior to MI, in one report among 22,227 MI patients from the Swedeheart registry [12]. In another report of 3129 women who experienced a first MI during follow-up in the Women’s Health Initiative study, 49% of women maintained low physical activity, or decreased their physical activity immediately after MI, compared to their activity prior to MI [13]. Several reasons have been suggested to explain this phenomenon, including a fear of exercising alone, far from possible help in case of symptom recurrence, and difficulties due to the side effects of secondary prevention medication [14]. Patients also report a lack of counselling about the frequency and intensity of exercise that would be appropriate for them. Some patients reportedly underestimate the gravity of their disease, and thus, do not perceive such a strong need to change their lifestyle [15], while others overestimate their level of disability, and are too afraid to undertake daily exercise alone, and yet others are highly motivated to change their daily habits after their infarction [9, 14].

Against this background, our study aimed to explore the intentions of patients hospitalized for AMI regarding attendance at cardiac rehabilitation and the pursuit of regular physical exercise at home. Using semi-structured interviews, we sought to understand the perceptions, expectations, barriers to and drivers of adherence to regular physical exercise at home after acute myocardial infarction.

## Methods

We performed a qualitative study using semi-structured interviews. The results are reported in accordance with the COREQ guidelines [16].

Eligible patients were those hospitalized for acute myocardial infarction at the Cardiology unit of a single university teaching hospital in Eastern France (University Hospital Besancon, France) from 10 November 2021 to 7 March 2022, and who were deemed eligible for cardiac rehabilitation by the treating physician. The researcher in charge of interviewing the patients (NS), attended the morning hand-over meeting, morning rounds, and the staff meeting every day to identify eligible patients and interview them before discharge, which takes place in the afternoon in our Department. The other main inclusion criteria were the ability to speak and understand French, and provision of informed consent.

Patients who accepted to participate were interviewed in their hospital room before discharge. No patient refused to participate. Interviews were recorded and transcribed for later analysis. We used an interview guide developed by a team of qualitative researchers (NS, FE, LP) based on knowledge of the discipline and a review of the literature. To construct the interview guide, we also consulted patients who had previously attended a residential cardiac rehabilitation programme, as well as one nurse manager and two cardiology residents from the Cardiology Department.

The interview guide covered the following points:

- The patient’s previous level of physical activity, prior to the AMI.
- What information the patients received about rehabilitation.
- Did the patient intend to attend rehabilitation in the residential cardiac rehabilitation centre or not.
- Expectations / fears about rehabilitation and the return to regular exercise, in the rehabilitation centre, and at home.

Interviews were performed by one researcher (NS, female, PhD candidate), who was not known to the patients and did not work in the Cardiology Department. Interview transcripts were analysed using thematic analysis [17]. Briefly, interviews were coded independently by 2 of the coauthors (NS, FE), to identify and categorize the different themes occurring in a cross-sectional manner across all interviews, (i.e. topics addressed at length by most, if not all individuals). The themes were classed as major themes (significant points that are of major importance and well developed by the participants) and secondary themes (less well developed by the participants). Meetings were held to triangulate data, harmonize and decide on the themes to be retained, and their regrouping into subject categories. Differences in interpretation were resolved by discussion and consensus. Interviews were conducted until data saturation was reached. Patients were informed that illustrative citations from their interview could be used to substantiate scientific publications (after translation), and all patients agreed to this.

We also administered the Global Physical Activity Questionnaire (GPAQ) questionnaire to all participants (Available at: https://www.who.int/publications/m/item/global-physical-activity-questionnaire). The GPAQ is a 16-item questionnaire developed by the World Health Organization for the surveillance of chronic disease risk factors. The questions are designed to estimate an individual’s level of physical activity in 3 domains, namely work, transport and leisure time, as well as the amount of time during which the individual is sedentary. Scoring was done according the WHO recommendations, and identified moderate-to-vigorous physical activity vs. sedentary behaviour. The average amount of daily physical activity was also estimated, by self-reporting from the patient.

## Results

Between 10 November 2021 and 7 March 2022, a total of 17 eligible patients were identified, of whom 15 were included. The majority were males (n = 13, 86%), median age 54 years (quartile (Q)1,=41, Q3 = 61 years). The characteristics of the study population are described in Table 1, as well as their self-reported level of physical activity prior to AMI, as assessed by the GPAQ. The average duration of the interviews was 15 min (range 10 to 21 min).

**Table 1:** Baseline characteristics of the study population with self-report duration of physical activity per day prior to infarction, and level of activity as assessed by the Global Physical Activity Questionnaire (GPAQ)

Pt	Sex	Age	Days since index MI	Profession	Self-estimated duration of physical activity per day pre-MI	Level of activity pre-MI as assessed by GPAQ
1	M	35	2	Builder	5h25min	High
2	M	50	3	Long-haul truck driver	26 min	Moderate
3	M	72	3	Retired	6 h	High
4	M	55	44	Mechanic on ski lifts	3h30min	High
5	M	40	3	Tractor mechanic	6h23min	High
6	M	67	2	Retired	1h42min	Low
7	M	41	34	Project manager	3h44min	High
8	M	61	1	Retired	2h52min	Moderate
9	M	57	2	On long-term sick leave	< 10 min	Low
10	F	61	2	Cleaner in a school	4h45min	High
11	M	75	2	Retired	5h30min	High
12	M	36	4	Unemployed	1h30	High
13	F	42	3	Cook	3h22min	Moderate
14	M	54	3	On long-term sick leave	1h00min	Moderate
15	M	43	4	Electrician	5h34min	High

MI, myocardial infarction; M, male; F, female. Age in years

**Fig. 1:**
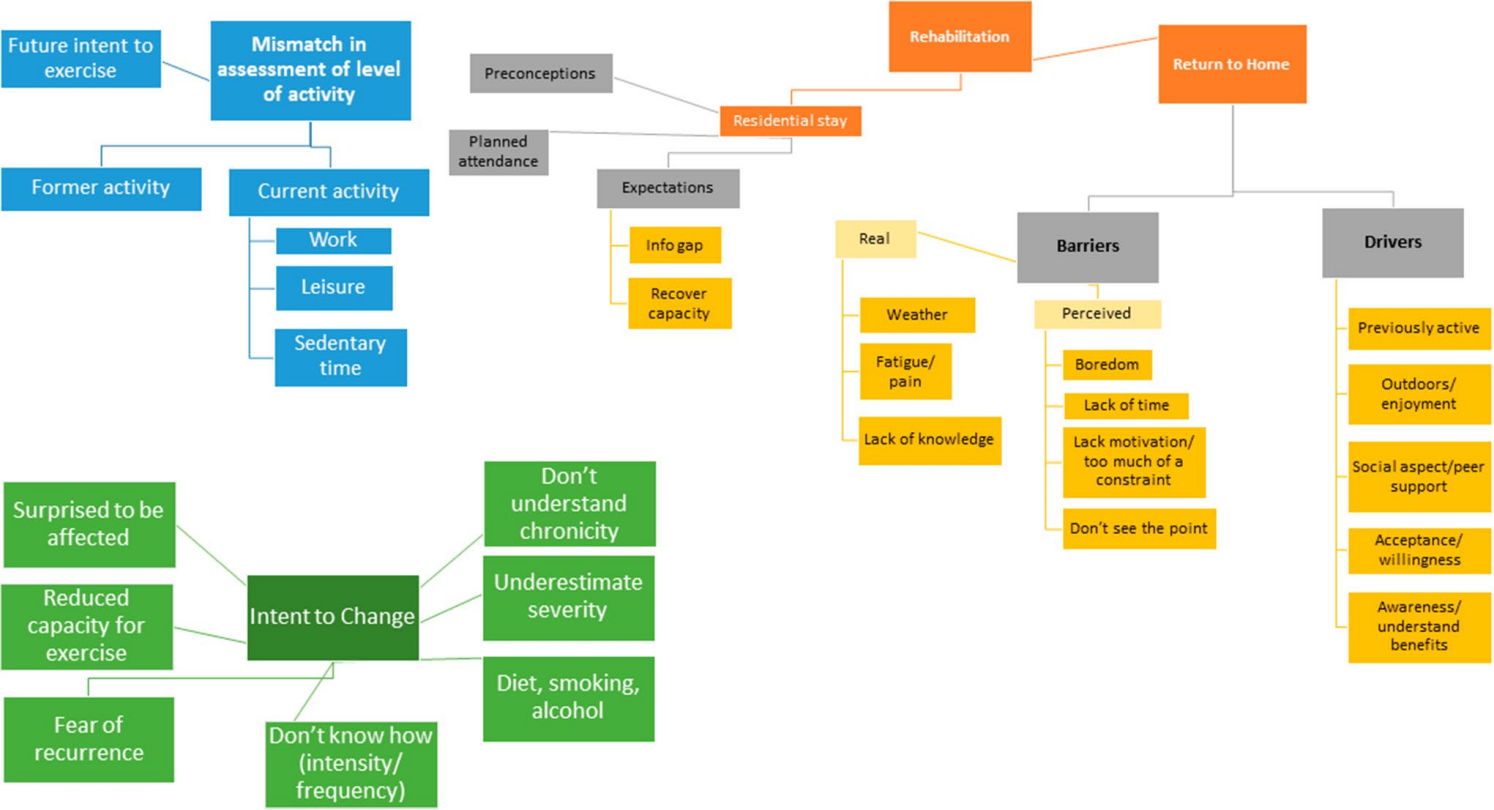
Conceptual framework of the main themes

Three major themes emerged from the analysis of the interviews, namely: [1] there is a mismatch between the patient’s perception of their level of activity and the actual level of physical activity as assessed by objective tools [2]. Cardiac rehabilitation is seen as a vector for information about the return to home after AMI [3]. Regarding the intention to change lifestyle, there is persistence and emergence of obstacles, drivers, fears and expectations. Each theme is described in detail below. A conceptual framework summarizing the themes is presented in Fig. 1.

### Theme 1: mismatch between perceived and actual level of physical activity

Regarding the actual level of physical activity, there was wide variability between patients, and a mismatch between what the patient reported, and the level of activity as calculated by the GPAQ score. For many patients, the level of activity they reported as “current” actually corresponded to sports practiced in the past. All the patients who reported that they currently engaged in regular sports activities also reported they had had quite an intensive level of sports activity in the past. The only patients who exercised regularly but without a history of intense sports activity were those who had been obliged to take up physical activity during a previous rehabilitation after an acute health event:

“*I had an accident with my back about ten years ago…. I did some physio, and now I do some movements 2 or 3 times a week*” (Patient 07FN).

The patients often reported a perceived level of exercise that was higher than the actual level as assessed by the GPAQ questionnaire. Participants who never engaged in intensive physical activity on a regular basis did not report being physically active at the time of the study, but they did have a tendency to overestimate their current physical fitness.

*“My job as a truck driver, it’s not what people think…. Those trucks weigh 50 ton, and they’re 18 metres long. It takes a lot of concentration and that requires a lot of energy!” (02CB – moderate level of physical activity as assessed by GPAQ score).*

*“I’m a cook, I’m standing up all the time” (13CA – moderate level of activity)*.

We also noted that the patients who had the least mismatch between perceived and assessed level of activity were those who had done a lot of sport when they were younger (either regular intensive sport, or competitive sport). Among these participants, some even had a tendency to underestimate their current level of physical activity:

*“Well, I don’t do intensive activity like I used to… I just do it for leisure, not to beat any world records” (04GG – vigorous level of physical activity)*.

“*When I was younger, I did more sport than now” (06LP – low level of activity)*.

*“My studies were long so I didn’t really do much sport any more, then I got married and well… you know, now it’s just for leisure” (10RC – high level of activity)*.

The majority of participants related their surprise at having suffered a myocardial infarction, and how quickly it happened. Most of these claimed to lead relatively healthy lifestyles, while others put the blame on smoking or stress, but no patient saw their MI as resulting from a lack of physical activity.

*“Well, really, it just kind of happened like that, because I never had any health problems really… I never had anything” (01CV)*.

*“I’m under a lot of stress… and, well…. I smoke, that doesn’t help either” (14VC)*.

*“I gave up smoking immediately” (07FN)*.

*“I have to stop smoking, reduce the alcohol and walk a bit more” (09NA)*.

Overall, the patients’ own estimation of their current level of physical activity is inaccurate, and they do not see physical activity as being strongly implicated in their disease process.

### Theme 2: the rehabilitation centre as a vector for information

#### Residential rehabilitation

The patients in this study were interviewed while still in hospital after their MI, and thus, had not had much time to gain perspective regarding the events befalling them. At the time of the interviews, the patients had only very recently been informed that they would be oriented to rehabilitation. Before being informed about their referral to rehabilitation, some of the patients did not know that such a programme existed, or had only a very vague idea of what rehabilitation entails.

*“That’s precisely the question I forgot to ask – isn’t that the place where people go to do gymnastics? Because if that’s what it is, I don’t want that. I want something clinical” (06LP)*.

For those who were aware of what rehabilitation entailed (informed via their entourage, for example), the majority had received positive feedback about rehabilitation, and therefore, were not averse to the idea of going there.

*“I’ve heard good things about that” (04GG)*.

Furthermore, they had some basic ideas about how residential rehabilitation centres work, and the fact that it entails tailored physical activity.

*“I think it’s like, medically assisted sport” (05VC)*.

Despite the recent announcement that they were being referred for rehabilitation, all the patients expressed a firm intention to attend the programme in a residential rehabilitation centre. The main reason cited was the need for guidance, principally concerning the appropriate level of physical activity. The patients explained that they needed to know when they could or could not do exercise, or indeed, in stronger terms, when they must do exercise, and when they must refrain from exercise. They clearly had informational needs regarding the return to (or initiation of) physical activity.

*“Yes, I’d be interested in going, so that someone will tell me … because if they let me go home now, and I don’t know what I’m supposed to be doing in terms of exercise…. If I go to rehabilitation and my heart doesn’t start beating too fast, they’ll have all the machines to tell me it’s OK” (13CA)*.

#### Information about home-based exercise

Contrary to residential rehabilitation, the patients had greater difficulty envisaging and articulating the return to home. This could be explained by the rapid onset of the MI, and the fact that the interviews were held very soon after admission and diagnosis. Most patients were nonetheless conscious of the fact that lifestyle changes would be necessary once they returned home, and that they would have to do regular exercise to reduce the risk of recurrence.

*“I don’t do any exercise, I don’t move at all. I’m going to have to change that. I understand that” (06LP)*.

*“I don’t see myself taking up physical activity without going to rehabilitation first… that won’t work” (07FN)*.

*“I know I’ll have to exercise every day” (06LP)*.

The participants unanimously reported an intention to pursue physical activity, or do more exercise, or take up exercise for those who did none, with the aim of preserving their health. Some underlined that the stay in rehabilitation would be useful to them to get informed, and better prepare their return to home.

*“I intend to do exercise. You have to be careful the first month, don’t go back to work or overdo it, and re-train my body to …eh…. take up activity” (03BJ)*.

*“Well, depending on the exercises they get me to do, I’ll be able to see whether I can adapt those, to something I can do outside of my home” (01CV)*.

*“I think I’ll have to take it easy at the beginning. I’m going to be very out of breath at the start” (04GG)*.

*“They’ll tell me more about it there, I presume” (02CB)*.

Overall, rehabilitation was viewed positively, mainly for its information potential, and as an intermediary step between the hospital and home.

### Theme 3: intent to change lifestyle: obstacles, drivers, fears, expectations

As for the return to home, when asked about a potential change to their lifestyle, many patients cited the early timing of the interview as an explanation for their inability to look so far ahead:

*“I haven’t thought about that yet. I might start thinking about that during next week” (03BJ)*.

*“I’ll see what the doctors say. I think they’re going to give me exercises to do” (01CV)*.

#### Emergence of obstacles, drivers, fears, and expectations related to the onset of disease

The acute cardiac event, such as infarction, was frequently cited as a major motivating factor for a change of lifestyle, to move towards a healthier mode of living, notably including more exercise.

*“Now I plan to walk for half an hour every day” (06LP)*.

*“I had a heart attack due to the nicotine” (04GG)*.

*“The doctor told me I had to move, not sit around doing nothing” (11GC)*.

In addition to taking up exercise, the participants who had deleterious lifestyle habits (such as smoking, drinking or poor diet) firmly stated their intention to change.

*“I’ve completely given up smoking, so normally, I shouldn’t get another heart attack because of that… and also, I might eat less fatty foods, you know, because I had high cholesterol too. But I have no fear …. It’s easy, you know” (04GG)*.

In the discourse of some participants, it became evident that the rapidity of management, and the efficacy of the care, returning them to an almost “normal” state of health, led them to underestimate the seriousness of the event, and the underlying disease.

*“It’s like as if nothing ever happened” (10RC)*.

This underestimation of the seriousness was reflected by the patients’ tendency to discuss it as an acute event, and not a chronic process, thereby reducing the impact of the infarction as a motivator for long-term lifestyle modifications.

*“Maybe after a while, if I feel better, I won’t do [the exercise] again, or maybe only once in a while…. I don’t know really” (13CA)*.

The patients’ overestimation of their level of physical activity, combined with the fact that the impact of rehabilitation is not immediately perceptible, resulted in some patients having a lower level of motivation for long-term adherence to the lifestyle modifications recommended by the physician.

*“When I had the knee operation, I went on the exercise bike at home to get my knee back in shape… but I could feel what I was doing, I knew why I was doing it, and that motivated me. But this time…. Well, I feel fine, and now I’m going to have to do all these things that seem to be quite useless to me…. I know that’s probably not true, but still, it’s less motivating, because I have other things to be doing” (11GC)*.

Two different points of view emerged with regard to the fear of recurrence during physical activity. On the one hand, some patients were afraid that they would not recognize, and might go beyond the new physical limits of their body:

*“It’s hard to know your limits” (07FN)*.

*“I don’t know…eh… what I can really do, in terms of effort” (13CA)*.

On the other hand, the second group of patients had no concerns about resuming exercise:

*“I think that having stents is absolutely not a problem, on the contrary… And besides, I seem to be fine” (04GG)*.

When asked about possible surveillance methods, such as telemonitoring of their heart rate, or using connected devices (e.g. smartphone), some patients welcomed the idea as reassuring and motivating, while others thought it would be restrictive and unhelpful.

*“It would be reassuring because in reality, it’s hard to be just …. let go, like that!”(07FN)*.

*“I’m not sure I’d be too enthusiastic about that idea” (02CB)*.

Finally, several participants reported a fear of being considered “too weak”, or of being unable to return to the daily activities performed prior to the MI. Accordingly, the strong desire to return to previous capacity was a motivating factor for many patients.

“Well, in the end, what I’m afraid of is that I’ll try to do something and realize that I’m no longer able. Or that it will happen me again, and I’d be saying to myself, F*** [expletive], I’m 35 and I’m done for, there’s nothing I can do about it. That’s what scares me the most” (01CV).

*“I don’t want to stay a vegetable, definitely not” (03BJ)*.

*“I really want to get back the level of fitness that I had before” (07FN)*.

#### Persisting obstacles, drivers, fears, and expectations, despite the onset of disease

Most of the obstacles to future performance of regular exercise cited by the participants were the same as those that had prevented them from doing exercise prior to their infarction, namely a lack of time, the weather, fatigue, pain, or a lack of motivation in the absence of a social dimension:

*“pffff [sighs]…. I’m on my own, it bores me more than anything else” (10RC)*.

*“I can’t walk any more because of my back” (09NA)*.

*“I like a quiet life, so it’s more of a constraint than anything else” (06LP)*.

*“Weekends are for resting. When I do too much…. Well, look what happens!” (02CB)*.

For patients who previously engaged in a lot of sports, adhering to the recommendations for regular exercise was not perceived as problematic, as they were “used to it”.

*“I don’t know how it’s going to work exactly, but I don’t mind cycling at all, I cycle a lot” (07FN)*.

Social support, ownership of sports equipment, and a suitable outdoor environment were all seen as factors that would facilitate regular exercise:

*“Often in the forest, because I live in the countryside, so there are plenty of tracks through the woods nearby” (15MA)*.

Regarding group activities, again, there were two schools of thought among the participants – firstly, those were reticent, because of the time constraints (*“I don’t know if I’ll have the time”)*, and secondly, those who find the group environment more motivating (*“I think it helps, to make you go there and do it. When you’re on your own, you think, yeah, I’ll do it tomorrow, but when there’s a group and they say, let’s meet at whatever time to go cycling, and you say, sure, that’s ok… yes, yes it definitely helps” (15MV)).*

Overall, the barriers to physical exercise reported by the patients were mainly those that had prevented from ever doing exercise before. The motivation stemming from an acute health event may not be sufficient for many patients to overcome their “lifestyle inertia” and change their habits durably, especially when they feel well and “normal” after acute treatment in the hospital.

## Discussion

This study explored the perceptions of patients with recent MI regarding cardiac rehabilitation and the return to (or initiation of) regular exercise. The main findings were a mismatch between the perceived level of exercise reported by the patients, and the actual level as assessed by the GPAQ; secondly, the informational needs of the patients, who reported that attending rehabilitation would help them to better apprehend their physical limits and be informed about what level of exercise is appropriate for them; and thirdly, a number of barriers to and drivers of regular exercise and lifestyle modification were cited by the participants.

Clearly, the patients’ perception of their lifestyle habits, and principally their level of exercise, influences their intention to take up exercise, and to pursue lasting lifestyle change incorporating more exercise. Their intentions to change were also impacted by their beliefs about their disease, and notably, motivation for lasting change may have been mitigated by the false impression that the acute health event was not too serious. Indeed, the fact that the patients felt well again very soon after their infarction, and could see little or no difference in their bodies, meant that they did not fully realize the gravity of the event in health terms. This gave them the impression that the MI was acute (and therefore “cured”), and not the manifestation of an underlying chronic process [15]. This impression could reduce the motivating impact of the MI as a driver of lifestyle change, and thereby, could reduce their motivation to adhere to recommendations for regular exercise. These results are in line with those of Coull & Pugh who found that the motivating effect of MI as a driver of lifestyle change waned over time, especially when other life stressors gained more importance, and the utility of exercise was no longer directly perceptible [14]. Similarly, it has previously been reported that efforts to instigate lasting lifestyle changes are more effective early in the disease course, including motivational interviewing or nursing interventions [18, 19]. This underlines again the importance of capitalizing on the period early after the acute event to implement rehabilitation and capitalize on patients’ early willingness to change.

Another interesting finding is the mismatch between the level of physical activity reported by the patients, and the actual level as assessed by an objective questionnaire. In reality, most patients were far from achieving the recommended level of exercise, in terms of both volume and intensity. Several patients believed that their profession provided enough physical activity, or that their leisure exercise met the recommended goals. Clearly, the beliefs of the patients were not in line with the reality of the WHO recommendations for 150 to 300 min of moderately intense aerobic activity, or at least 75 to 150 min of intense aerobic activity [20]. In this regard, there is a compelling need to emphasize the information and education component during rehabilitation, to provide simple and quantifiable goals for exercise, starting as soon as possible after the acute event [21]. However, when interpreting the self-reported level of activity, it should be noted that there is conflicting data regarding the accuracy of the GPAQ as an estimator of activity, particularly in comparison to objective measures with wearable devices, such as accelerometers or actigraphs. Indeed, Wanner et al. reported in a cross-sectional study of 354 participants that the GPAQ showed only fair-to-moderate validity for the assessment of actual physical activity, and that its estimates were 2.8 times higher than activity measured with accelerometer data [22]. Conversely, Laeremans et al. reported that GPAQ estimates were significantly lower for exercise across the range of moderate to vigorous intensity [23]. Finally, a meta-analysis including 148 studies found no clear trend in the degree of correlation between self-reported and directly measured physical activity, with low-to-moderate correlations overall (mean 0.37 ± 0.25, range − 0.71 to 0.98) [24]. In that same study, in a meta-analysis of 74 studies comparing self-reported versus directly measured physical activity, 60% of the mean differences indicated that self-reported physical activity estimates were higher than those measured by direct methods [24]. The over-estimation of self-reported exercise in our study is in line with these data, and may result from patients having perceived the exercise to be more intense than it actually was. Under-estimation may arise if patients fail to consider the activities of daily living, such as housework or gardening, when estimating their physical activity levels. Overall, the conflicting data suggest that self-reporting may not be an accurate estimation of exercise; nevertheless, the French version of the GPAQ has been demonstrated to provide acceptable (albeit limited) reliability and validity for the measurement of physical activity and sedentary time in a French adult population [25].

The patients in our study also had unmet informational needs regarding the appropriate behaviour once they returned home, and were unsure of whether they could exercise, and if so, how much and how often. Undoubtedly, this is why they were all enthusiastic about attending rehabilitation, in the hope that they would get answers to their questions there. A corollary of this is that the patients obviously do not consider residential rehabilitation as a therapeutic intervention, requiring compliance in the same way as drug therapy [8], but rather, as an opportunity to get information for what to do when they return home. In the study by Coull & Pugh, the authors reported that the patients still had the same informational gaps, even after rehabilitation, with a lack of advice and clear guidelines about the volume and intensity of exercise [14]. In this regard, medical prescriptions for adapted exercise therapy by general practitioners, introduced in France in 2017, could be a useful solution. This prescription enables patients with long-term disease to receive personalized coaching for physical activity adapted to their disease, their physical fitness and their medical risk. In this paradigm, exercise is seen as a therapeutic intervention to maintain or improve health, of similar value to drugs and devices. Exercise prescriptions must be adapted to the patient’s individual risk profile to achieve maximum efficacy at acceptable safety, in the same way as drug therapy [26]. This may also help to allay any fears patients may harbour about the safety of home-based exercise, especially since it has been shown in a systematic review that deaths or hospitalizations are very rare during home-based cardiac rehabilitation, with an estimated incidence rate of severe adverse events of 1 per 23,823 patient-hours of exercise [27].

The peer-support provided by group exercise activities may also promote more diligent attendance [28]. This is line with a previous report indicating that the routine of a structured class, as well as recovery-specific self-efficacy are necessary to sustain long-term adherence to cardiac rehabilitation [29].

Personalizing exercise programmes is all the more attractive and important when one considers the wide heterogeneity in fitness levels among the patients prior to their MI. It has been reported that patients with poor baseline capacity are more likely to drop out of rehabilitation [7]. A range of structural factors have also been associated with non-adherence to rehabilitation, including low income, low social support, or long travel times to rehabilitation centres [30]. Our study also highlights the link between prior habits in terms of exercise, and intent to pursue physical activity at home. It has previously been reported that the emotional response following MI can be harnessed by healthcare professionals and patients alike to prompt lifestyle changes towards risk-reducing behaviours [14, 18]. However, healthcare professionals should be wary of the potential for the impact of the event to wane, as life returns to normal, and the emotional impact of MI is forgotten.

In practical terms, this study has some implications for practice and research. Firstly, as discussed above, self-reported measures of physical activity should be interpreted with caution in both practice and research. Secondly, the barriers to accessing and adhering to cardiac rehabilitation should be addressed. At the individual level, this means impressing upon patients the importance of rehabilitation for their future health, and the mortality benefits to be gained from increased physical activity after MI [12, 13]. Providing or financing transport to rehabilitation facilities could overcome travel barriers [10]. At the level of the physicians or department, standardized protocols for referrals to rehabilitation could ensure that all eligible patients receive the appropriate referral [10], while at national level, providing reimbursement for cardiac rehabilitation through national social security programmes could also help overcome cost barriers that prevent some citizens from attending [31]. Finally, remote delivery of cardiac rehabilitation programmes that patients can perform at home has been shown to be affordable, accessible, and reliable as an alternative means of achieving health-promoting behaviours [32]. Such e-health solutions minimize time and geographic barriers, and empower patients to acquire knowledge and skills, and develop and focus on personal goals and action plans [33].

This study has some limitations. Firstly, the interviews were performed early after the event, and therefore, the cognitive understanding of the consequences of coronary artery disease may have been influenced by the emotional response to experiencing an acute health event. In addition, the intentions expressed by the participants regarding rehabilitation were all hypothetical since none of them had been to rehabilitation yet. It would be interesting to collect their opinions again after a minimum time interval (perhaps 6 months), to see whether their perspectives about rehabilitation and exercise had changed. Despite the inclusion of a wide diversity of patients from a large, university teaching hospital, we cannot rule out potential selection bias, since patients ineligible for rehabilitation were not included in this study. Finally, there is also potential for recall bias in the estimation of physical activity using the GPAQ, since patients are asked to report on activity levels in the past.

## Conclusion

This study shows that patients who suffer from acute myocardial infarction often have an overly optimistic estimation of their level of physical activity, and there is a mismatch between reported levels and measured levels of activity, as assessed by the GPAQ. Even close to discharge after infarction, patients have persisting informational needs regarding appropriate levels of physical activity, and many patients see cardiac rehabilitation as a means of obtaining information, and not as a therapeutic intervention. The obstacles to and drivers of exercise prior to suffering from MI are still reported after the event. The power of a health event such as MI should be harnessed by patients and healthcare professionals to encourage patients with coronary artery disease to undertake lifestyle modifications for the long term, in order to mitigate cardiovascular risk.

## Data Availability

Interview transcripts are confidential, but anonymized data can be made available upon written request to the corresponding author.
